# Understanding Patient Perspectives on Digital Smoking History Data Collection

**DOI:** 10.1016/j.chpulm.2025.100180

**Published:** 2025-05-19

**Authors:** Kayla C. Jones, Lauren E. Kearney, Emily Jansen, Nicholas Cordella, Hasmeena Kathuria, Katrina Steiling

**Affiliations:** a Evans Center for Implementation & Improvement Sciences, Department of Medicine, Boston University Chobanian & Avedisian School of Medicine, Boston, MA; b The Pulmonary Center, Boston University Chobanian & Avedisian School of Medicine, Boston, MA; c Department of Quality and Patient Safety, Boston Medical Center, Boston, MA; d Center for Tobacco Research and Intervention, Department of Medicine, School of Medicine and Public Health, University of Wisconsin-Madison, Madison, WI

**Keywords:** digital outreach, electronic health record, lung cancer screening, patient experience, patient-generated health data, qualitative research, smoking history

## Abstract

**Background:**

Lung cancer screening with an annual low-dose CT scan is recommended by the US Preventive Services Task Force for high-risk patients based on age (50-80 years of age) and smoking history (≥ 20 pack-years). Inaccurate smoking history data in the electronic health record (EHR) pose a challenge to identifying eligible patients. Digital strategies to collect patient-generated health data (PGHD) are a potential solution to elicit complete smoking histories directly from patients to then integrate into their EHR.

**Research Question:**

How do patients perceive and experience the use of digital outreach strategies to collect smoking history data to determine lung cancer screening eligibility?

**Study Design and Methods:**

As part of a quality improvement initiative, semistructured qualitative interviews were completed with a diverse group of patients who had received a request to complete a digital smoking history survey. Rapid analytical methods were used.

**Results:**

We completed 20 interviews with patients who did (n = 9) and did not (n = 11) complete the digital smoking history survey. Participants described varied preferences for digitally self-updating their smoking histories. Four themes emerged regarding barriers to uptake including (1) participants prefer to update their health record with a clinician, (2) technologic barriers influence participant engagement with digital PGHD collection strategies, (3) multiple options for collecting smoking frequency are needed to align with a variety of behaviors, and (4) participants have mixed perceptions of the value of accurate and updated EHR smoking data.

**Interpretation:**

Our findings highlight the need to address barriers to collection of digital PGHD to optimize reach and uptake. Using PGHD to obtain an updated and accurate smoking history has important implications, and incorporating patient education, strategies to overcome technologic barriers, and multiple metric options to collect smoking history may improve use.

**Take-Home Points**

**Study Question:** How do patients perceive and experience the use of digital outreach strategies to collect smoking history data to determine lung cancer screening eligibility?

**Results:** Participants described varied preferences for digitally self-updating their smoking histories, and 4 major themes emerged regarding barriers to uptake that included patient preferences, technological barriers, smoking frequency collection not aligning with behaviors, and mixed perceptions regarding the value of accurate and updated electronic health record smoking data.

**Interpretation:** Using patient-generated health data to obtain an updated and accurate smoking history has important implications, and collection barriers could be addressed by incorporating patient education, implementing strategies to overcome technological barriers, and using multiple metric options to collect smoking history.

Cigarette smoking is an important risk factor for numerous diseases and the leading cause of lung cancer, which accounts for the highest rate of cancer-related deaths.^1^^,^^2^ Screening and early detection of lung cancer are critical for improving outcomes.^3^ The current US Preventive Services Task Force recommendation for lung cancer screening (LCS) is an annual low-dose CT scan for people between 50 and 80 years of age, with ≥ 20 pack-year smoking history, and who are either currently smoking or have quit in the previous 15 years.^2^

However, LCS rates in the United States remain extremely low (approximately 16% of eligible patients)^4^ because of a variety of barriers. For example, identifying eligible patients requires complete and accurate smoking history documentation to calculate the pack-year metric (number of years smoking × packs per day). However, the smoking history documented in the electronic health record (EHR) is often inaccurate, outdated, and incomplete.^5^, ^6^, ^7^, ^8^, ^9^ Although the unstructured data in the EHR (eg, documented as free text in notes) and new technology (eg, natural language processing) may provide opportunities for more reliable and complete smoking histories, deployment of these solutions is limited in current practice.^10^^,^^11^ Thus, we explored an alternative solution using digital strategies to collect patient-generated health data (PGHD) aimed at eliciting an accurate smoking history.^12^

Our previously conducted pragmatic randomized trial to evaluate the effectiveness of digital PGHD approaches (ie, text message, portal-based surveys) to collect smoking history data found that digital PGHD approaches are a feasible way to engage patients and capture complete smoking histories.^13^ We now report on our qualitative study findings conducted to elicit patient perspectives and experiences with updating their smoking history through digital PGHD collection approaches with the goal of informing future iterations of PGHD capture.

## Study Design and Methods

This qualitative study was conducted at a large urban safety net hospital in New England. The hospital is a tertiary care academic medical center that serves a diverse population. Participants were recruited from the patient list that was generated for the previously described pragmatic randomized trial.^13^ The inclusion criteria for eligibility included the following: history of tobacco use (active or previous), age 50 to 80 years, and English as a preferred language. The digital smoking history survey (e-Appendix 1) queried patients regarding current cigarette smoking status, cigarette smoking start date, quit date, and cigarette packs per day. The digital smoking history survey design has been previously described.^13^ From the pragmatic randomized trial patient list, we randomly sampled participants with specific attention to recruitment of those who did and did not complete the portal or text message-based smoking history survey (hereafter, digital smoking history survey) for broad representation. Participants were contacted via telephone, provided a description of the interview process, and invited to participate. Participants that agreed to an interview were read the consent form, provided verbal consent, and were then scheduled for a telephone interview. This project was deemed to be exempt by the Boston Medical Center and Boston University Medical Campus institutional review board. We followed the consolidated criteria for reporting qualitative research checklist (e-Appendix 2).^14^

A semistructured interview guide (e-Appendix 3) was developed by the study team, guided by the integrated Promoting Action on Research Implementation in Health Services,^15^ a framework used for implementing evidence-based practices in health care. The interview questions were designed to elicit perspectives regarding participants’ experiences and preferences with a self-reported digital smoking history survey and to identify actionable suggestions to improve the process for future iterations of PGHD collection.

One-on-one interviews were conducted by 2 female interviewers that included a medical anthropologist (K. C. J.) and a pulmonologist with qualitative experience (L. E. K.). Interviewers had no prior relationships with participants. Interviews were completed between July and October 2023. The average interview time was 11 minutes (range, 6-21 minutes). Interviews were conducted via telephone and were audio-recorded. On completion of the interview, participants were compensated with a $20 debit card to thank them for their time. Data collection was complete once thematic saturation was achieved.^16^

The audio-recorded interviews were professionally transcribed, and identifying information was removed. The analyst team, consisting of 2 analysts (K. C. J. and L. E. K.), used rapid analytical methods.^15^^,^^16^ The lead analyst (K. C. J.) designed a templated summary table in Microsoft Word (Microsoft Inc) based on the interview guide that was then reviewed and edited with the study team. The analysts independently summarized key points from transcripts in the summary table template, noting illustrative quotes. The summaries were aggregated according to survey completion status and entered into the summary table matrix. Once complete, the lead analyst reviewed the summary matrix table and synthesized the summaries into themes with representative quotes, and the second analyst (L. E. K.) reviewed and revised themes. The study team (E. J., N. C., H. K., and K. S.) further reviewed and revised the themes until consensus was achieved. Themes were compared between survey completion status to identify overlapping and differing themes.

## Results

### Participant Characteristics

Participant demographics and characteristics are provided in Table 1. A total of 20 participants completed interviews, including 9 participants who completed the digital smoking history survey and 11 who did not. The average age of participants was 60.5 years of age. Most of the participants (70%) were Black and 20% were Hispanic.

**Table 1:** Participant Characteristics

Characteristic	Value
Age, y	60.5 (50-73)
Responder vs nonresponder	
Nonresponders	11 (55.0)
Responders	9 (45.0)
Race	
Black	14 (70.0)
White	3 (15.0)
Other	2 (10.0)
Declined	1 (5.0)
Ethnicity	
Not Hispanic, Latino, Latina, Latinx, or of Spanish or Latin American origin	16 (80.0)
Hispanic, Latino, Latina, Latinx, or of Spanish or Latin American origin	4 (20.0)
Gender	
Woman	12 (60.0)
Man	8 (40.0)

A total of 194 patients were contacted for interviews, and 31 eligible patients declined to participate. Data are presented as mean (range) or No. (%).

### Qualitative Themes

Participants described varied preferences for digitally self-updating their smoking histories. Four themes regarding barriers to participation in digital PGHD strategies to self-update smoking histories were identified: (1) participants prefer to update their health record with a clinician, (2) technologic barriers influence participant engagement with digital PGHD collection strategies, (3) multiple options for collecting smoking frequency are needed to align with smoking behaviors, and (4) participants have mixed perceptions of the value of accurate and updated EHR smoking data.

#### Participants Prefer Clinicians Update EHR

Participants had mixed perceptions about the optimal process for obtaining and maintaining an accurate smoking history in the EHR. However, most participants reported a preference to update their medical record with their clinician. Participants’ reasoning included having an established relationship with their clinician and viewing it as their clinician’s responsibility. One participant noted, “I'd rather be one-on-one with my primary care doctor … [b]ecause it’s the privy of my doctor and that’s who’s got to take care of me and that’s who should be responsible for it” (Complete 26). In addition to perceiving updates to the EHR as the clinician’s responsibility, participants who did not complete the digital smoking history survey thought that smoking history assessments would be done more expeditiously and more accurately if completed by a clinician: “I thought I would forget to do [the survey] or I wouldn’t do it. I think that doctors would write down things correctly at the time of my visit and all of that, whereas I’d be like, ‘oh, I forgot about that’” (Incomplete 401).

#### Technological Barriers Affect PGHD Engagement

In addition to participants having an overall preference to update their medical record with a clinician, many also cited low digital proficiency as a central driver for this preference. One person noted, “I’d work with the humans. I’m not too good with computers so I’d rather have a person to deal with it. …I’m not that great with those computer systems…” (Incomplete 50).

Many participants who did not complete the digital smoking history survey reported limited access to digital technologies that influenced their ability to complete the digital smoking history survey: “I don’t use a cell phone. This is a house phone [I’m talking on right now]” (Incomplete 952).

Participants described varied digital access, technologic proficiency, and personal preferences. For example, participants expressed concern about potentially missing both text- and/or portal-based messages. Some participants who did not complete the digital smoking history survey expressed concern about important health information being lost due to text message fatigue: “I get so many text messages and sometimes if it’s from [the hospital] and it’s around when I have an appointment, because I’ll get like four texts about my appointment that’s coming up. …I don’t answer them all…” (Incomplete 401). Other participants worried about potentially missing portal-based messages because they do not routinely log in to their account. One participant said, “But a text is probably better because I don’t have appointments every month and so I don’t check [the portal]. The month I know I got [an] appointment, I will go into [the portal]” (Incomplete 1083). Another noted, “I think a text message would do better because I rarely open up MyChart. I’m just getting into MyChart, but I very rarely open it up…" (Complete 982).

When probed about the reasons for not logging into their portal account, patients discussed how they perceived the portal as more difficult to use compared with text messages. One participant explained their experience with the portal as, “…[Y]ou gotta go and open [the portal] up and—you’re not really gonna open it up, but if you get a text, people look at their texts” (Complete 982).

#### Multiple Options for Collecting Frequency

Participants provided feedback on how to report the amount smoked (ie, average number of cigarettes smoked per day). Some participants reported nondaily smoking patterns which are not easily captured in EHR data fields: “…[S]ome people like myself smoke occasionally, I’ll smoke one cigarette one day, I'll smoke three cigarettes the next, and another day I’ll smoke no cigarettes…” (Incomplete 401). Participants highlighted the need to have multiple options to report their smoking behavior that include cigarettes per day, packs per day, and a metric that encapsulates infrequent smoking to fit a range of behaviors: “… [A]sk the question about race, and you’re like, ‘Well, I don’t fit anywhere in these categories. There should be more options.’ So, the same with like cigarettes” (Complete 837). Individuals that smoked regularly preferred to report cigarette use as packs per day: “I personally remember the packs per day because it was a finite amount constantly” (Complete 911). Whereas others, particularly those who had variable smoking patterns, preferred to report cigarettes per day: “[I prefer] cigarettes per day…[b]ecause I know how much I smoke of cigarettes a day…I don’t smoke a whole pack a day. I smoke probably like three or four [cigarettes]” (Incomplete 423).

#### Importance of Accurate Smoking History

We found mixed perceptions among participants regarding the accuracy of the smoking history recorded in the EHR. Some thought that their smoking history was accurately updated in the EHR: “…[T]hey know what’s in my records about the smoking” (Complete 44). Others acknowledged a disconnect between their actual smoking histories and those captured in the structured EHR field: “…I just had a procedure done the other day. The doctors thought that I was still smoking a pack a day and I’m like, ‘no, I don’t smoke anymore.’… ‘[I]t says you're a full [on] smoker’” (Incomplete 50).

Some saw the value of PGHD approaches to updating the smoking history especially as a way to help the medical team: “…[Self-updating the medical record] would really probably help out. …You’re helping out your doctor [and] helping yourself” (Incomplete 248). Others viewed an updated, accurate smoking history as a source of motivation to stop smoking because it allows them to track their cessation progress: “I’m still in the process of cutting myself out completely from smoking…I think that keeping an accurate record helps me see that I am slowly stopping” (Complete 1037). However, some participants did not see the value of updating the records through PGHD approaches because they regularly attend clinical appointments: “[Self-updating] my medical history don’t really matter to me, I go to the doctor pretty often” (Incomplete 952).

Although the connection of an accurate smoking history and tobacco cessation was clear to participants, participants did not readily recognize the connection between an accurate smoking history and LCS eligibility. For example, 1 participant stated, “I’m not really sure how [smoking history and lung cancer screening] works. …I don’t think [updating my smoking history] would have much of an impact on my life” (Incomplete 50).

Finally, we found that among those who had stopped smoking, the importance of an updated smoking history was not clear. They expressed limited familiarity with health risks that persist after quitting smoking, not connecting how their smoking data in the EHR are still relevant to their health. Participants who had stopped smoking emphasized how long it has been since they quit smoking, distancing themselves from potential health consequences: “[I’m] no longer a smoker and I quit somewhere around four years ago. So, it’s a little bit beyond the pale at this point” (Complete 911). This was also true regarding LCS because many people who had quit smoking did not appreciate a potential ongoing need for screening: “I think if my smoking history was more recent [LCS would be important].”

## Discussion

This qualitative study explored patient experiences and preferences on providing an updated smoking history through the digital PGHD collection strategies used in our pragmatic trial to improve smoking history completeness.^13^ This study was designed to enhance our understanding of the reasons patients did and did not complete a digital survey to update their smoking history to determine LCS eligibility. Additionally, this study provides valuable insights into the potential utility and barriers of digital strategies for collecting smoking history with PGHD. Our analytical goal was to understand how this approach could be improved and implemented as a promising strategy to collect complete smoking history and improve LCS implementation.^5^, ^6^, ^7^, ^8^

Although our previous quantitative analysis suggests that PGHD is an effective way to collect smoking histories,^13^ this study demonstrates potential barriers to using digital strategies to collect PGHD that might limit uptake of these approaches, and illustrates opportunities to improve acceptability (Table 2). Overall, participants in our study preferred discussing their smoking behaviors with their clinician because of their established relationship and a perception that updating EHR data was a clinician’s responsibility. This highlights the need to improve patient education on how PGHD approaches can be used in clinical decision-making and how PGHD can complement, rather than replace, face-to-face clinical interactions.^12^ Additionally, improving patient education provides the opportunity to empower patients to use and contribute to the EHR and to partner with their clinicians.^17^^,^^18^

**Table 2:** Perceived Barriers to PGHD Implementation and Possible Solutions

Participant Identified Barriers	Proposed Solutions^a^
Preference for clinicians to update the patient’s health record: overall, participants prefer to update the EHR with a clinician.	Clarify and provide reassurance to patients that PGHD is used in addition to but not in place of 1-on-1 clinical interactions.^11^^,^^16^^,^^17^
Technological barriers: • Patients experience technology barriers that impact their ability to engage with digital PGHD collection strategies (eg, no cell phone). • Portal messages may present additional barriers due to having to log in to the portal account.	Provide a variety of modality options when collecting PGHD (eg, text message, portal, phone call). Health systems could systematically collect patient communication preference in the EHR and pair this with the appropriate modality. Health systems could compile patient reminders, text messages, and portal messages into a digest sent at predefined intervals to prevent alert fatigue.
Multiple options for collecting frequency: patients have unique and varied preferences for reporting their smoking use, influenced in part by their frequency and duration of smoking. This poses challenges to capturing smoking histories in a uniform manner.	When collecting PGHD, offer patients multiple metric options to report their smoking use. Collect sufficient data to be able to translate results back to discrete data fields in the EHR (ie, if patient prefers to report cigarettes/d, divide by 20 to get packs/d).
Importance of accurate smoking history: • Patients were surprised that data in their EHR (eg, smoking history) may not be accurate. • Mixed perception on the value of updating the EHR themselves. • Importance of having updated and accurate smoking data in the EHR is unclear to patients. • Participants were unaware of the connection between an updated and accurate smoking history for LCS eligibility.	Provide patient education on the potential benefits of updating their data outside of office visits using digital tools. For example, updated smoking data provided asynchronously to an office visit can help their clinician make better-informed decisions about screening options. Engage patients in smoking cessation treatment when collecting smoking history data (eg, allow patients to track their smoking reduction progress,^19^, ^20^, ^21^ provide education on smoking cessation and benefits of quitting smoking). Provide patient education on lung cancer risk at the time of the survey. Tailor messaging based on patient answers to earlier survey questions (ie, if a patient self-reports they have prior tobacco use, highlight that patients 50-80 y of age who fomerly smoked with ≥ 20 pack-year smoking history that have quit smoking within the past 15 y are eligible for LCS).

EHR = electronic health record; LCS = lung cancer screening; PGHD = patient-generated health data.

a Solutions proposed by the authors.

Although our pragmatic trial evaluating the effectiveness of portal questionnaires compared with SMS text message-based surveys showed that patients were more likely to respond to text messaging,^13^ this qualitative analysis revealed barriers to both digital strategies. Identified barriers include a lack of comfort or limited access to digital communication technologies (eg, no cell phone or internet access), infrequent use of the EHR patient portal, and too high frequency of other text messages. These barriers suggest a need for multiplicity of collection methods for digital PGHD strategies. Our findings of technology access issues and limited comfort with technology are supported by the literature, which shows that although there are many benefits of using technology to engage older patients in their care, there are also significant barriers to uptake and continued use.^22^^,^^23^ This is important to consider when implementing strategies to capture smoking history data for LCS, given the older age demographic (50-80 years of age).

Our results highlight the need for various methods for patients to report their smoking history because different smoking behaviors can affect clinical care. Future PGHD collection efforts should offer patients multiple options to report their smoking use because smoking behaviors and patterns impact clinical care, including determining eligibility for LCS and options for tobacco treatment. This is especially important for equity in LCS and smoking cessation treatment because findings from prior studies have shown that Black populations have high rates of lung cancer incidence and mortality but are more likely to report nondaily smoking.^19^^,^^24^^,^^25^ It is essential to provide Black adults with tobacco treatment and evaluate their LCS eligibility in a way that fully accounts for nondaily and low-intensity smoking.

One potential approach is to integrate PGHD strategies with tools to monitor changes in smoking behavior because some participants viewed tracking their smoking behavior as a source of motivation. Similar to other studies,^20^^,^^21^^,^^26^ participants in our study liked the idea of reporting smoking frequency as a way to track progress of smoking reduction. Smoking reduction is a strategy for individuals unable or unwilling to quit smoking that aims to lessen adverse health effects and represents an important step toward cessation.^27^ This suggests an opportunity to integrate cessation resources and tracking into smoking history data collection as a way to encourage smoking cessation and improve capture of accurate and up-to-date smoking histories.

To enhance digital methods for collecting smoking history data, it may be beneficial to integrate patient education during the data collection process, even for those who have quit smoking. During our qualitative interviews, participants reported limited knowledge of LCS. However, those who received a brief explanation of LCS subsequently recognized its benefits (eg, early detection of lung cancer, gaining more health information) and understood the importance of an accurate smoking history for determining LCS eligibility, including for people who had already quit smoking. Although there are many reasons for this low baseline knowledge,^28^, ^29^, ^30^, ^31^ patient eagerness to hear about LCS during our study suggests a potential solution may lie in integrating LCS education into smoking history survey data collection. Based on the results of our qualitative study, we propose the education should focus on highlighting who is eligible for LCS, with an emphasis on ongoing risks for lung cancer even if someone has quit smoking. Given the need for shared decision-making in LCS, the education should also stress the importance of talking with a doctor about if LCS is right for the patient. However, the optimal approaches to disseminating educational information about LCS and how eligibility is determined (eg, having an updated and accurate smoking history in the medical record) require additional investigation. Combining patient education with digital data collection could increase survey completion rates and enhance patient motivation to quit smoking and access LCS.

Our study has limitations. First, there was a time delay between when patients received the smoking history survey and the date of the interview. The average time from the survey to the interview was 7.5 months due to delays in institutional review board review and approval of the study, which may have limited participants’ ability to recall specific details about their experience. Despite this limitation, we were able to garner insightful information about their preferences related to collecting smoking history through digital PGHD strategies. Second, our sample was limited to participants with English documented as their preferred language in the EHR and who were engaged in care at an urban safety net hospital. Although the sample was diverse in terms of race/ethnicity, future work that focuses on the experience of non-English-speaking patients, patients in other health care settings, and patients in rural settings engaging with digital strategies to collect smoking history may yield additional insights into barriers and facilitators to the use of PGHD. Nonetheless, our findings offer valuable insights into the patient experience with engaging in PGHD collection aimed at updating smoking history data. Third, our study focused on the collection of cigarette use history which can be leveraged for determination of LCS eligibility. However, the approaches which we evaluate in this qualitative study may inform collection of other data that has been difficult to accurately collect and document in the EHR.^32^ This includes PGHD of complete nicotine use screening (eg, chewing tobacco, smokeless tobacco) and other smoking history (eg, electronic cigarette use, vaping, hookah or shisha use, marijuana smoking).

## Interpretation

In this qualitative study of the patient experience with collecting smoking history through digital PGHD collection strategies, we uncovered important findings to improve future PGHD initiatives. Key insights included the importance of the clinician’s role in collecting medical information, technological barriers to engaging with PGHD collection, and unclear benefit of self-updating smoking history based on smoking status. Despite these preferences and barriers, our pragmatic randomized trial found a high level of completeness suggesting that PGHD collection via text message survey is feasible and effective.^13^ For widespread implementation, our qualitative results highlight the need for modality options that engage patients and are easy to use. Our data further suggest that linking digital smoking history surveys with education on lung cancer risk and LCS may increase patient engagement. Technological barriers must also be addressed. Future work should explore strategies to address the barriers to engagement in digital PGHD strategies identified in our study, and to assess their effect on accurately and consistently collecting smoking histories to facilitate LCS.

## Funding/Support

The authors have reported to *CHEST Pulmonary* that no funding was received for this study.

## Financial/Nonfinancial Disclosures

The authors have reported to *CHEST Pulmonary* the following: H. K. received NIH funding unrelated to this work. None declared: K. C. J., L. E. K., E. J., N. C., and K. S.

## Supplementary Data

e-Online Data

## References

[1] Siegel R.L., Miller K.D., Jemal A. (2020). Cancer statistics, 2020. CA Cancer J Clin.

[2] US Preventive Services Task Force, Krist A.H., Davidson K.W. (2021). Screening for lung cancer: US Preventive Services Task Force Recommendation Statement. JAMA.

[3] The National Lung Screening Trial Research Team (2011). Reduced lung-cancer mortality with low-dose computed tomographic screening. N Engl J Med.

[4] American Lung Association Lung cancer key findings, American Lung Association website. https://www.lung.org/research/state-of-lung-cancer/key-findings.

[5] Polubriaginof F., Salmasian H., Albert D.A., Vawdrey D.K. (2018). Challenges with collecting smoking status in electronic health records. AMIA Annu Symp Proc.

[6] Steiling K., Loui T., Asokan S. (2020). Age, race, and income are associated with lower screening rates at a safety net hospital. Ann Thorac Surg.

[7] Modin H.E., Fathi J.T., Gilbert C.R. (2017). Pack-year cigarette smoking history for determination of lung cancer screening eligibility. comparison of the electronic medical record versus a shared decision-making conversation. Ann Am Thorac Soc.

[8] Kukhareva P.V., Caverly T.J., Li H. (2022). Inaccuracies in electronic health records smoking data and a potential approach to address resulting underestimation in determining lung cancer screening eligibility. J Am Med Inform Assoc.

[9] Fedewa S.A., Kazerooni E.A., Studts J.L. (2020). State variation in low-dose computed tomography scanning for lung cancer screening in the United States. JNCI J Natl Cancer Inst.

[10] Ruckdeschel J.C., Riley M., Parsatharathy S. (2023). Unstructured data are superior to structured data for eliciting quantitative smoking history from the electronic health record. JCO Clin Cancer Inform.

[11] Wang L., Ruan X., Yang P., Liu H. (2016). Comparison of three information sources for smoking information in electronic health records. Cancer Inform.

[12] Adler-Milstein J., Nong P. (2019). Early experiences with patient generated health data: health system and patient perspectives. J Am Med Inform Assoc.

[13] Kearney L.E., Jansen E., Kathuria H. (2024). Efficacy of digital outreach strategies for collecting smoking data: pragmatic randomized trial. JMIR Form Res.

[14] Tong A., Sainsbury P., Craig J. (2007). Consolidated criteria for reporting qualitative research (COREQ): a 32-item checklist for interviews and focus groups. Int J Qual Health Care.

[15] Harvey G., Kitson A. (2016). PARIHS revisited: from heuristic to integrated framework for the successful implementation of knowledge into practice. Implement Sci.

[16] Guest G., Bunce A., Johnson L. (2006). How many interviews are enough?: an experiment with data saturation and variability. Field Methods.

[17] Bhattad PB, Pacifico L (2022). Empowering patients: promoting patient education and health literacy. Cureus.

[18] Hickmann E., Richter P., Schlieter H. (2022). All together now – patient engagement, patient empowerment, and associated terms in personal healthcare. BMC Health Serv Res.

[19] Skolnick S., Cao P., Jeon J., Meza R. (2023). Contribution of smoking, disease history, and survival to lung cancer disparities in Black individuals. JNCI Monogr.

[20] Gowarty M.A., Kung N.J., Maher A.E., Longacre M.R., Brunette M.F. (2020). Perceptions of mobile apps for smoking cessation among young people in community mental health care: qualitative study. JMIR Form Res.

[21] Vilardaga R., Rizo J., Kientz J.A., McDonell M.G., Ries R.K., Sobel K. (2016). User experience evaluation of a smoking cessation app in people with serious mental illness. Nicotine Tob Res.

[22] Wilson J., Heinsch M., Betts D., Booth D., Kay-Lambkin F. (2021). Barriers and facilitators to the use of e-health by older adults: a scoping review. BMC Public Health.

[23] Buyl R., Beogo I., Fobelets M. (2020). e-Health interventions for healthy aging: a systematic review. Syst Rev.

[24] Arauz R.F., Mayer M., Reyes-Guzman C., Ryan B.M. (2022). Racial disparities in cigarette smoking behaviors and differences stratified by metropolitan area of residence. Int J Environ Res Public Health.

[25] Haiman C.A., Stram D.O., Wilkens L.R. (2006). Ethnic and racial differences in the smoking-related risk of lung cancer. N Engl J Med.

[26] Heffner J.L., Vilardaga R., Mercer L.D., Kientz J.A., Bricker J.B. (2015). Feature-level analysis of a novel smartphone application for smoking cessation. Am J Drug Alcohol Abuse.

[27] Yoo J.E., Han K., Shin D.W. (2022). Effect of smoking reduction, cessation, and resumption on cancer risk: a nationwide cohort study. Cancer.

[28] Gressard L., DeGroff A.S., Richards T.B. (2017). A qualitative analysis of smokers’ perceptions about lung cancer screening. BMC Public Health.

[29] Carter-Harris L., Ceppa D.P., Hanna N., Rawl S.M. (2017). Lung cancer screening: What do long-term smokers know and believe?. Health Expect.

[30] Carter-Harris L., Hermann C.P., Schreiber J., Weaver M.T., Rawl S.M. (2014). Lung cancer stigma predicts timing of medical help–seeking behavior. Oncol Nurs Forum.

[31] Saab M.M., Noonan B., Kilty C. (2021). Awareness and help-seeking for early signs and symptoms of lung cancer: a qualitative study with high-risk individuals. Eur J Oncol Nurs.

[32] Winden T.J., Chen E.S., Wang Y., Sarkar I.N., Carter E.W., Melton G.B. (2015). Towards the standardized documentation of e-cigarette use in the electronic health record for population health surveillance and research. AMIA Summits Transl Sci Proc.

