# Supplementary material for: Understanding Patient Perspectives on Digital Smoking History Data Collection
Source: CHEST Pulm. 2025 May 19;3(3):100180. doi: 10.1016/j.chpulm.2025.100180 (PMC13418914; doi:10.1016/j.chpulm.2025.100180)
Supplement: e-Online Data [file mmc1.docx]

**Supplemental Materials**

| **Portal Questionnaire** | **Text Survey** |
| --- | --- |
| **Please select all that apply to describe**  **your tobacco use history**:   - I stopped smoking cigarettes - I currently smoke cigarettes - I have never smoked cigarettes   **Individuals currently smoking only:**  **How often do you smoke?**   - Every Day - Some Days   **How old were you when you started**  **smoking?**  **Individuals who formerly smoked only:**  **How old were you when you stopped**  **smoking?**  **How many cigarettes do/did you**  **usually smoke in a day? (1 pack = 20**  **cigarettes)**  **What is the most cigarettes you have**  **ever smoked in a day?**  **If you use something other than**  **cigarettes, please select all that apply:**   - I smoke cigars/cigarillos - I use e-cigarettes/vape every day - I use e-cigarettes/vape some days - I use chew - I smoke tobacco pipes - I use snuff - I have never used any tobacco products | **Please select the answer that describes your tobacco use history:**   - I stopped smoking cigarettes - I currently smoke cigarettes - I have never smoked cigarettes   **How old were you when you started smoking?** Please enter a number between 1-100 or enter “0” if you never  smoked.  **How many cigarettes do/did you usually smoke in a day? (1 pack = 20 cigarettes)** Please enter a number between 1-100 or enter “0” if you never smoked.  **If you stopped, how old were you when you stopped smoking?** Please enter a number between 1-100 or enter “0” if you are a current smoker or never smoked. |

**File 1**: Digital Smoking History Portal Questionnaire and Text Survey Questions

**File 2:** Consolidated criteria for reporting qualitative studies (COREQ): 32-item checklist^13^

| **No. Item** | **Guide questions/description** | **Reported on Page #** |
| --- | --- | --- |
| **Domain 1: Research team and reﬂexivity** |  |  |
| *Personal Characteristics* |  |  |
| 1. Inter viewer/facilitator | Which author/s conducted the interview or focus group? | Page 4 |
| 2. Credentials | What were the researcher’s credentials? E.g. PhD, MD | Page 1 |
| 3. Occupation | What was their occupation at the time of the study? | Page 4 |
| 4. Gender | Was the researcher male or female? | Page 4 |
| 5. Experience and training | What experience or training did the researcher have? | Page 1 and 4 |
| *Relationship with participants* |  |  |
| 6. Relationship established | Was a relationship established prior to study commencement? | Page 4 |
| 7. Participant knowledge of the interviewer | What did the participants know about the researcher? e.g. personal goals, reasons for doing the research | Page 3 and 4 |
| 8. Interviewer characteristics | What characteristics were reported about the inter viewer/facilitator? e.g. Bias, assumptions, reasons and interests in the research topic | Page 3 |
| **Domain 2: study design** |  |  |
| *Theoretical framework* |  |  |
| 9. Methodological orientation and Theory | What methodological orientation was stated to underpin the study? e.g. grounded theory, discourse analysis, ethnography, phenomenology, content analysis | Page 4 |
| *Participant selection* |  |  |
| 10. Sampling | How were participants selected? e.g. purposive, convenience, consecutive, snowball | Page 3 and 4 |
| 11. Method of approach | How were participants approached? e.g. face-to-face, telephone, mail, email | Page 3 |
| 12. Sample size | How many participants were in the study? | Page 4 and Table 1 |
| 13. Non-participation | How many people refused to participate or dropped out? Reasons? | Table 1 |
| *Setting* |  |  |
| 14. Setting of data collection | Where was the data collected? e.g. home, clinic, workplace | Page 3 |
| 15. Presence of non-participants | Was anyone else present besides the participants and researchers? | Page 4 |
| 16. Description of sample | What are the important characteristics of the sample? e.g. demographic data, date | Page 4 and Table 1 |
| *Data collection* |  |  |
| 17. Interview guide | Were questions, prompts, guides provided by the authors? Was it pilot tested? | Page 3 and 4 and Supplemental file 2 |
| 18. Repeat interviews | Were repeat interviews carried out? If yes, how many? | No, inferred on page 4 |
| 19. Audio/visual recording | Did the research use audio or visual recording to collect the data? | Page 4 |
| 20. Field notes | Were ﬁeld notes made during and/or after the interview or focus group? | No, inferred on page 4 |
| 21. Duration | What was the duration of the inter views or focus group? | Page 4 |
| 22. Data saturation | Was data saturation discussed? | Page 4 |
| 23. Transcripts returned | Were transcripts returned to participants for comment and/or correction? | No, inferred on page 4 |
| **Domain 3: analysis and ﬁndings** |  |  |
| *Data analysis* |  |  |
| 24. Number of data coders | How many data coders coded the data? | Page 4 |
| 25. Description of the coding tree | Did authors provide a description of the coding tree? | No |
| 26. Derivation of themes | Were themes identiﬁed in advance or derived from the data? | Page 4 |
| 27. Software | What software, if applicable, was used to manage the data? | Page 4 |
| 28. Participant checking | Did participants provide feedback on the ﬁndings? | No, inferred on page 4 |
| *Reporting* |  |  |
| 29. Quotations presented | Were participant quotations presented to illustrate the themes/ﬁndings? Was each quotation identiﬁed? e.g. participant number | Page 5 to 9 |
| 30. Data and ﬁndings consistent | Was there consistency between the data presented and the ﬁndings? | Yes, there was.  Page 5 to 9 |
| 31. Clarity of major themes | Were major themes clearly presented in the ﬁndings? | Yes they were.  From page 5 to 9 |
| 32. Clarity of minor themes | Is there a description of diverse cases or discussion of minor themes? | Discussion of major and minor themes  From page 5 to 10 |

**File 3:** Interview guide

**Introduction:**

We are trying to improve and update patient smoking history in the medical record at [institution name]. To help use with this, we would like to hear about your experience competing or not complete a smoking history survey.

**Interview Guide for Patients Who Completed Smoking History Survey**:

1. You recently received a survey asking for you to update your smoking history. What are your thoughts on updating your smoking history? Do you think it is important?
   1. In what ways, if any, do you think reporting/updating your smoking history will have a positive impact on your life/health?
   2. What about any negatives?
2. What are your thoughts on self-updating your medical history?
   1. Do you have a preferred way of updating your medical record? Probe for reasoning
3. Do you remember receiving the message about updating your smoking history?
4. Tell me about your experience with completing the smoking history survey
   1. What were your reasons for completing the survey?
   2. What were the factors that made it easy to complete?
   3. What were the factors that made it difficult to complete?
   4. Did you receive a reminder to complete it? How was the reminder helpful or not helpful?
5. When reporting how frequently you smoke, do you find it easier to recall the average number of cigarettes smoked per day, the packs per day, or another metric?
   1. Probe for reasoning
6. How would you change the delivery of the survey to make it more likely that you will respond?
   1. What aspects of the survey would make you less likely to respond?
   2. Why is that your preference? Probe for contextual factors
7. What emotions and feelings did you experience when filling out the survey?
8. Has anyone ever discussed lung cancer screening with you or have you heard about lung cancer screening?
   1. If no, provide brief description of LCS then probe for benefits
      1. Description: Lung cancer screening is usually a once-a-year CT scan to check for early signs of lung cancer in people who smoke or used to smoke and who are at higher risk for lung cancer. There are pluses and minuses for having this test and it’s important that patients talk to their doctor to find out if screening is right for them
   2. If yes, what do you know about lung cancer screening
   3. Do you believe there are benefits of lung cancer screening? If so, what are the benefits?
      1. What about risks or concerns that you have?
   4. Do you think it’s important?
   5. Do you think there is a connection between updating your smoking history and lung cancer screening?
      1. Clarify why/why not they think there is/isn’t a
9. IF ELIGIBLE FOR LCS: Have you had a lung cancer screening CT since completing the survey?
   1. If not, why? Has anyone talked with you about lung cancer screening? Are there any barriers (e.g., work, childcare, transportation, insurance, etc.)? Or concerns (e.g., radiation exposure, etc.)? Do you plan on scheduling one if you qualify?
   2. If yes, can you talk about your experience (did you encounter any issues, was it easy to schedule, did you get the results, etc.)

**Interview Guide for Patients Who Did Not Complete Smoking History Survey:**

1. You recently received a survey asking for you to update your smoking history. What are your thoughts on updating your smoking history? Do you think it is important?
   1. In what ways, if any, do you think reporting/updating your smoking history will have a positive impact on your life/health?
   2. What about any negatives?
2. Has anyone ever discussed lung cancer screening with you or have you heard about lung cancer screening?
   1. If no, provide brief description of LCS then probe for benefits
      1. Description: Lung cancer screening is a once-a-year CT scan to check for early signs of lung cancer in people who smoke or used to smoke and who are at higher risk for lung cancer. There are pluses and minuses for having this test and it’s important that patients talk to their doctor to find out if screening is right for them
   2. If yes, what do you know about lung cancer screening
   3. Do you believe there are benefits of lung cancer screening? If so, what are the benefits?
      1. What about risks or concerns that you have?
   4. Do you think it’s important?
   5. Do you think there is a connection between updating your smoking history and lung cancer screening?
      1. Clarify why/why not they think there is/isn’t a connection
3. What are your thoughts on self-updating your medical history?
   1. Do you have a preferred way of updating your medical record? Probe for reasoning
4. When reporting how frequently you smoke, do you find it easier to recall the average number of cigarettes smoked per day, the packs per day, or another metric?
   1. Probe for reasoning
5. Do you remember receiving a message to update your smoking history?
6. What were the factors that made it difficult for you to complete the survey?
   1. What were the factors that made it difficult to complete?
      1. Probe about the delivery (i.e., that it was sent via text/MyChart, the wording of the message, etc.)
      2. Probe about other contextual factors (time, forgot, phone plan/access to internet, etc.)
   2. Did you receive a reminder to complete it?
      1. Probe how the reminder was helpful or not helpful
7. How would you change the delivery of the survey to make it more likely that you will respond?
   1. What aspects of the survey would make you less likely to respond?
